# Identification and Functional Characterization of FMN2, a Regulator of the Cyclin-Dependent Kinase Inhibitor p21

**DOI:** 10.1016/j.molcel.2012.12.023

**Published:** 2013-03-07

**Authors:** Kayo Yamada, Motoharu Ono, Neil D. Perkins, Sonia Rocha, Angus I. Lamond

**Affiliations:** 1 Centre for Gene Regulation and Expression, College of Life Sciences, University of Dundee, Dow Street, Dundee DD1 5EH, UK; 2 Institute for Cell and Molecular Biosciences, Medical School, Newcastle University, Framlington Place, Newcastle Upon Tyne NE2 4HH, UK

## Abstract

The ARF tumor suppressor is a central component of the cellular defense against oncogene activation in mammals. p14ARF activates p53 by binding and inhibiting HDM2, resulting, inter alia, in increased transcription and expression of the cyclin-dependent kinase inhibitor p21 and consequent cell-cycle arrest. We analyzed the effect of p14ARF induction on nucleolar protein dynamics using SILAC mass spectrometry and have identified the human Formin-2 (FMN2) protein as a component of the p14ARF tumor suppressor pathway. We show that FMN2 is increased upon p14ARF induction at both the mRNA and the protein level via a NF-κB-dependent mechanism that is independent of p53. FMN2 enhances expression of the cell-cycle inhibitor p21 by preventing its degradation. FMN2 is also induced by activation of other oncogenes, hypoxia, and DNA damage. These results identify FMN2 as a crucial component in the regulation of p21 and consequent oncogene/stress-induced cell-cycle arrest in human cells.

## Highlights

► Proteomic analysis reveals that ARF induces FMN2 ► ARF, DNA damage, and hypoxia induce FMN2 transcription in a p53-independent manner ► FMN2 promoter is negatively regulated by NF-κB and E2F1 ► FMN2 regulates p21 protein levels by forming a complex and preventing its degradation

## Introduction

The ARF tumor suppressor initiates the cellular response to aberrant oncogene activation through binding to and inhibiting the activity of Hdm2/Mdm2, the E3 ubiquitin ligase for p53 (Sherr, 2001; Vousden, 2002). As such, upon ARF induction, p53 can escape from degradation and activate transcription of its target genes. These include proapoptotic genes such as puma and noxa (Zilfou and Lowe, 2009) and cell-cycle inhibitors such as p21 (Zilfou and Lowe, 2009).

A high percentage of human leukemia and melanoma patients have ARF mutations (Curtin et al., 2005; Goldstein et al., 2007; Soufir et al., 2004). Furthermore, the ARF locus is found hypermethylated (and hence silenced) in a great number of human cancers (Badal et al., 2008; D’alessandro et al., 2002). Genetic studies have shown that ARF deletion promotes tumor development with high frequency (Sherr, 2001). Moreover, p53 action as a tumor suppressor is severely impaired in the absence of ARF (Christophorou et al., 2006; Efeyan et al., 2006). However, genetic and biochemical studies on p53 and ARF pathways showed there are also ARF tumor suppressor pathways that are p53 independent (Chen et al., 2009; Rocha et al., 2003, 2005; Wadhwa et al., 2002; Weber et al., 2000).

ARF accumulates in nucleoli during oncogene activation and either inhibits cell-cycle progression or promotes apoptosis through both p53-dependent and p53-independent mechanisms (Rocha et al., 2003, 2005). One of the p53-independent functions of ARF is the regulation of ribosome biogenesis in the nucleolus (Sherr, 2001).

The nucleolus is a subnuclear organelle in which rRNAs are transcribed, processed, and assembled with ribosomal proteins into ribosome subunits (Boisvert et al., 2007; Granneman and Baserga, 2004). However, recent studies also suggested that the nucleolus is not only the site of ribosome subunit biogenesis but also is associated with additional biological functions, e.g., cell-cycle regulation, stress responses, and human disease (Boulon et al., 2010b; Boyd et al., 2011; Pederson, 2011; Pederson and Tsai, 2009). Interestingly, studies on the rates of protein turnover in human nucleoli using pulse SILAC showed that p14ARF was one of the nucleolar proteins with the fastest rate of turnover (Lam et al., 2007).

The function of p14ARF in nucleoli is still not fully characterized. Furthermore, mechanistic aspects of ARF-mediated tumor suppression independent of p53 are relatively unknown. To address these questions, we performed an unbiased screen for proteomic changes in the nucleolus following p14ARF induction. Here we report the characterization of a component in the p14ARF tumor suppressor pathway, called FMN2. We find that FMN2 is induced by p14ARF at the transcriptional level, independent of p53, via a NF-κB-dependent mechanism. Importantly, FMN2 is required for stable protein expression of the cell-cycle inhibitor p21. FMN2 is necessary and sufficient for increasing p21 protein expression via a mechanism that involves the inhibition of protein degradation.

## Results

### Dynamic Change of Nucleolar Proteins during ARF Induction

To identify ARF-mediated changes in nucleoli, we performed a quantitative analysis of alterations to the nucleolar proteome following induction of p14ARF expression. For this we used two model human cell systems allowing inducible p14ARF expression that have been extensively characterized by us, and others (Llanos et al., 2001; Rocha et al., 2003, 2005). NARF2 cells are derived from the osteosarcoma cell line U2OS, which has the p14ARF gene promoter methylated and hence silenced. NARF2 cells possess an exogenous, IPTG-inducible p14ARF construct. In addition, we also used NARF2-E6 cells, which are analagous to the NARF2 cells, but in addition express the HPV protein E6. E6 continually targets p53 for degradation and as such renders the NARF2-E6 cells nonfunctional for p53 (Rocha et al., 2003, 2005).

**Figure 1:**
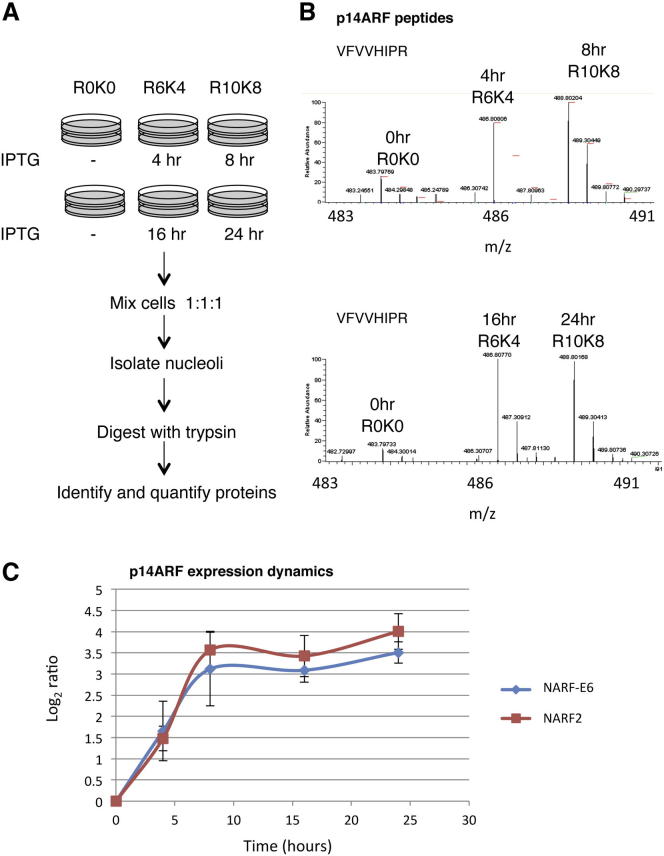
Determination of Nucleolar Protein Dynamics (A) The proteomes in three cell populations are encoded by incorporation of stable isotope derivatives of arginine (SILAC method). Cells are metabolically labeled with Arg0, Arg6, and Arg10 for at least five cell doublings and are then treated with IPTG for 0, 4, and 8 hr or 0, 16, and 24 hr to induce p14ARF, respectively. Cells are mixed and nucleoli purified and analyzed by mass spectrometry. The analysis is repeated three times with a common zero point. (B) Spectra of peptides of p14ARF, indicating increasing amounts of p14ARF recruited to the nucleolus after IPTG treatment. (C) Dynamic profile of p14ARF. The y axis is in units of normalized log_2_ change of p14ARF. Graph depicts mean and standard deviation from a minimum of three independent experiments. See also Figure S1.

Using these model human cell systems, we have analyzed ARF-induced nucleolar protein dynamics using SILAC mass spectrometry (Figure 1A) (Andersen et al., 2002, 2005; Boisvert et al., 2011; Lam et al., 2007). To confirm that the SILAC culture medium is compatible with these cell systems, we determined the G1, S, G2, and M populations of NARF2 cells grown both in normal culture medium and in SILAC medium (see Figures S1A and S1B online). This showed that the SILAC medium has little or no effect on NARF2 cell growth (Figures S1A and S1B). We also determined the quality of nucleoli purified from NARF2 cells (Figure S1C), and confirmed independently using immunofluorescence microscopy the nucleolar accumulation of p14ARF protein following IPTG induction (Figure S1D).

As a further control, we verified by MS analysis the presence of p14ARF peptides in nucleoli following induction with IPTG (Figure 1B). This revealed 86.8% sequence coverage of the p14ARF protein (data not shown). By performing a time course induction of p14ARF, we compared the dynamic change of p14ARF expression in both NARF2 and NARF-E6 cells. p14ARF protein levels increased in both cell lines after induction with IPTG, as expected (Figure 1C). We also compared the mass spectrometry data with signal intensity from fluorescence microscope-based live-cell imaging for GFP-tagged p14ARF. This revealed a similar increase in p14ARF expression levels as judged by both MS and microscopy-based quantitation methods (Figure S1E), further validating our analysis. Similar results were also obtained for other nucleolar proteins, including NPM1 and fibrillarin (FBL) (Figure S1F).

**Figure 2:**
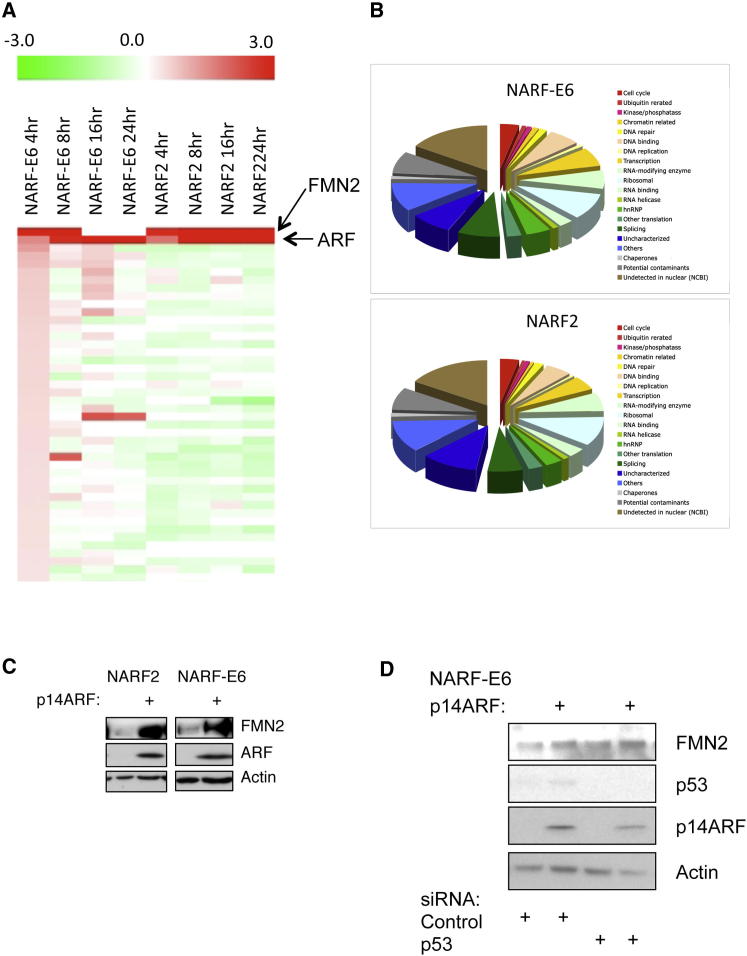
Dynamic Profiles of Nucleolar Proteins (A) Hierarchial clustering of top 5% of 3,500 proteins using fold change data. (B) Distribution pattern of proteins in each cell line. (C) NARF2 and NARF-E6 cells were harvested after 24 hr with or without IPTG induction and immunoblotted with FMN2, ARF, and actin antibodies. (D) NARF-E6 cells were transfected with control or p53 siRNA oligonucleotides prior to p14ARF induction for 24 hr. Whole-cell lysates were analyzed by western blot for the levels of the indicated proteins. See also Figure S2.

The mass spectrometry data identified changes in the relative levels of thousands of nucleolar proteins following p14ARF induction. The top 5% of proteins showing the largest relative change in abundance in the nucleolus are shown (Figure 2A). The majority of these proteins showed decreased levels in the nucleolus following p14ARF induction. Interestingly, the protein distribution pattern in NARF2 and NARF2-E6 was very similar, indicating that most, if not all, of these changes are independent of p53, at least in this model cell system (Figure 2B and Figure S2).

### Identification of an ARF-Induced Protein, FMN2

The MS analysis identified that in particular the Formin-2 (FMN2) protein was highly induced by ARF in a p53-independent manner. There is relatively little information on the function of the FMN2 protein, particularly in human cells, but reported roles include modulation of cytokinesis (Katoh and Katoh, 2004; Leader et al., 2002). However, to the best of our knowledge, no association of FMN2 with either p14ARF or p53 has previously been reported. Due to the dynamics and level of FMN2 induction observed by mass spectrometry, we decided to investigate the significance of this finding for downstream effects of p14ARF pathways.

Given the absence of previous studies on human FMN2 protein function, few reagents were available. We therefore cloned full-length human FMN2 cDNA (Figure S3, see the Experimental Procedures) and developed specific antibodies (Figure 2C and Figure S4). The anti-FMN2 antibodies detected FMN2 protein both by immunofluorescence microscopy and by protein blotting, with the signal specifically reduced following siRNA-mediated knockdown of FMN2, but not after treatment of cells with control siRNAs (Figures S4A–S4C). Protein blot analysis using these antibodies further showed that FMN2 levels increased after induction of p14ARF in both p53-positive and -negative cell lines, confirming the previous MS data (Figure 2C, Figures S4A and S4B). To further rule out any p53 dependency, siRNA-mediated depletion of p53 in NARF-E6 was performed. The data show that p53 is not required for p14ARF-mediated induction of the FMN2 protein (Figure 2D).

### ARF Upregulates FMN2 at the Transcriptional Level

**Figure 3:**
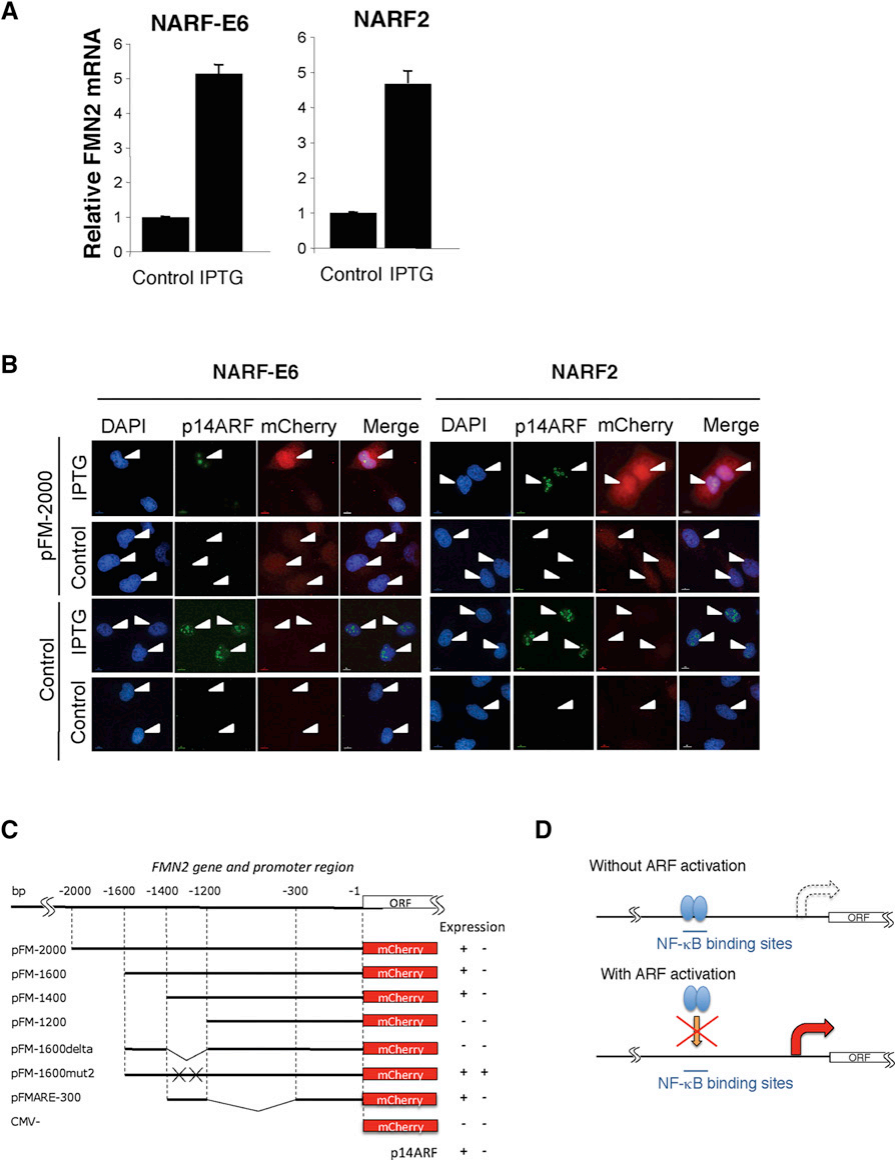
FMN2 Is Transcriptionally Upregulated by p14ARF Independently of p53 (A) Total RNA from NARF2 and NARF-E6 cells was harvested 24 hr after addition of IPTG. Following cDNA synthesis, qPCR was performed using FMN2-specific primers. Actin was used as a normalizing gene. Graph depicts mean and standard deviation from a minimum of three independent experiments. (B) NARF2 and NARF-E6 cells were transfected with control or FMN2 promoter constructs as indicated prior to IPTG induction. Twenty-four hours later, cells were fixed and analyzed by microscopy. (C and D) Schematic diagram summarizing FMN2 promoter analysis in NARF2 and NARF-E6 cells. See also Figure S3, Figure S4, and Figure S5.

To determine the mechanism behind increased FMN2 protein levels after induction of p14ARF, we next analyzed FMN2 mRNA levels by qPCR. This revealed that ARF induction increases FMN2 transcript levels (Figure 3A). In addition, northern blot analysis also demonstrated an increase in FMN2 transcript levels following ARF induction (Figure S5A). Furthermore, microarray analysis showed that FMN2 mRNA levels were highly increased following ARF induction (Figure S5B). In contrast, addition of IPTG to normal U2OS cells did not result in any significant change in FMN2 levels (Figure S5C). These data indicate that ARF regulates FMN2 expression either at the transcriptional level, or at the level of RNA stability.

To determine whether transcription of the FMN2 gene is regulated by ARF, we investigated if the activity of the FMN2 promoter was responsive to ARF induction. To do this, we cloned a 2 kbp genomic region from upstream of the FMN2 ORF, containing the predicted FMN2 promoter, into plasmid mCherry-N1, which possesses a truncated CMV promoter. This assay allows direct visualization of FMN2 promoter activity in expressing cells, at the single-cell level, based on detection of mCherry by fluorescence microscopy. In addition, a smaller 1 kbp region of this promoter was also cloned in the same construct. When transfected into either NARF2 or NARF2-E6 cells, addition of IPTG and hence ARF induction increased mCherry expression in both cell lines (Figures 3B–3D, Figures S5D and S5E). The negative control plasmid, which does not include any upstream genomic FMN2 promoter region, did not show mCherry expression either with or without ARF induction (Figures 3C and 3D, Figures S5D and S5E).

The previous results suggest that the FMN2 promoter has an ARF responsive element in the −1 to −2,000 bp region. Further analysis compared the ability of different sequences within this region of the promoter to support ARF-dependent induction of mCherry expression. For example, deletion of sequences from −2,000 up to −1,400 bp did not prevent ARF-mediated induction of mCherry. On the other hand, deletion up to −1,200 bp abolished ARF responsiveness of this promoter (Figure 3C, Figures S5D and S5E).

The promoter analysis revealed that a fragment corresponding to the DNA sequence between −1,400 and −1,200 of the FMN2 promoter was necessary and sufficient for ARF-mediated induction of mCherry (Figure 3C, Figures S5D and S5E). Closer inspection of the sequence within this 200 bp minimal fragment revealed overlapping putative binding sites for the transcription factors NF-κB and E2F1 (Figure S3, bold). Given the previous connection reported between ARF and NF-κB (Rocha et al., 2003, 2005), we tested for the involvement of these binding sites by mutating the two NF-κB sites in the FMN2 promoter constructs and repeating the analysis. Interestingly, mutation of the NF-κB sites resulted in constitutive expression of mCherry from the FMN2 promoter (Figure 3C, Figures S5D and S5E). We infer that NF-κB binding to the FMN2 promoter represses transcription of the FMN2 gene. Taken together, these results suggest that ARF may modulate NF-κB function to control FMN2 promoter activity (Figure 3D).

### ARF Upregulates FMN2 at the Transcriptional Level by Inhibiting NF-κB and E2F1

**Figure 4:**
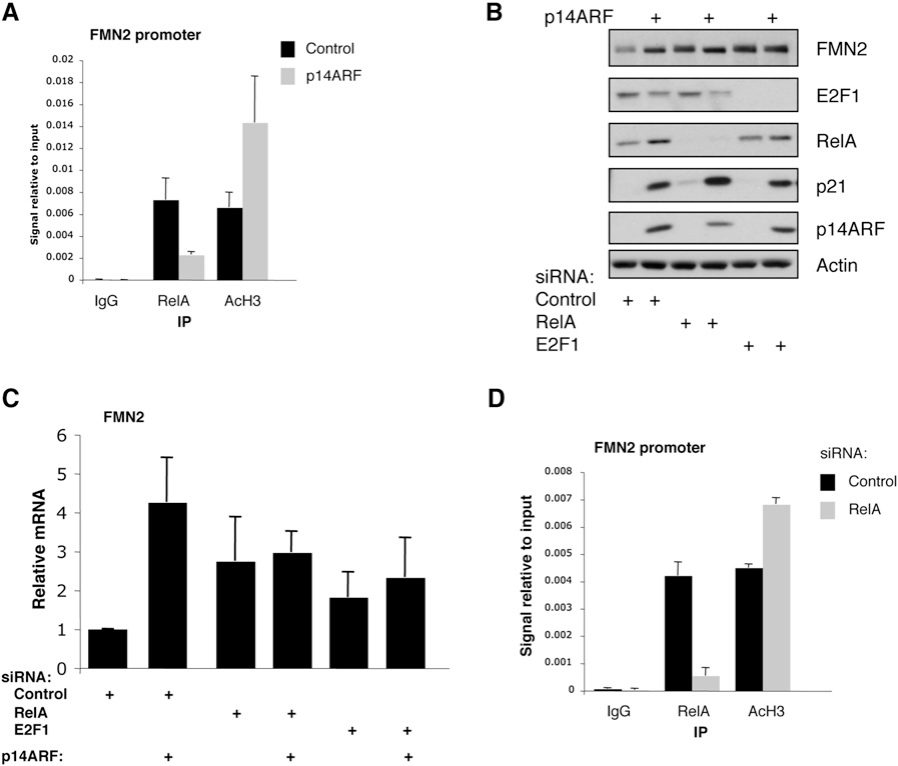
FMN2 Expression Is Repressed by NF-κB and E2F1 (A) NARF2 cells were induced or not with IPTG prior to crosslinking and lysis. Chromatin immunoprecipitation was performed using anti-RelA and anti-AcH3 antibodies, with rabbit IgG used as a control. qPCR was used to measure relative promoter occupancy levels compared to input material. Graph depicts the mean and standard deviation of a minimum of three independent experiments. (B) NARF2 cells were transfected with siRNA oligonucleotides for NF-κB/RelA or E2F1 prior to IPTG treatment for 24 hr. Whole-cell lysates were analyzed by western blot for the levels of the indicated proteins. (C) Cells were treated as in (B), but total RNA was extracted. After cDNA synthesis, qPCR analysis was performed for the levels of FMN2. Graph depicts mean and standard deviation from a minimum of three independent experiments. (D) U2OS cells were transfected with siRNA oligonucleotides for NF-κB/RelA prior to crosslinking and lysis. Chromatin immunoprecipitation was performed using anti-RelA and anti-AcH3 antibodies, with rabbit IgG used as a control. qPCR was used to measure relative promoter occupancy levels compared to input material. Graph depicts the mean and standard deviation of a minimum of three independent experiments. See also Figure S6.

The FMN2 promoter analysis revealed a possible role for NF-κB and E2F1 in the regulation of FMN2 by ARF. To validate these findings, we performed chromatin immunoprecipitation analyses on the FMN2 promoter, using NF-κB/RelA antibodies. We also used anti-AcH3 antibodies as a marker for active transcription. Under basal conditions we could detect NF-κB/RelA binding to the FMN2 promoter (Figure 4A). However, upon ARF induction, the amount of NF-κB/RelA present at the promoter was reduced (Figure 4A). Furthermore, this reduction in NF-κB/RelA binding was accompanied by an increase in the levels of AcH3 present in this region of the promoter, consistent with NF-κB/RelA acting as a transcriptional repressor of this gene. To determine if the results obtained with the promoter occupancy assay were reflected in the levels of FMN2 protein and mRNA, siRNA depletion of either NF-κB/RelA or E2F1 was performed, either with or without ARF induction in NARF2 cells. As seen before, ARF induction resulted in increased levels of both FMN2 protein (Figure 4B) and mRNA (Figure 4C and Figure S6). Interestingly, when either NF-κB/RelA or E2F1 was depleted, constitutive high levels of FMN2 protein and mRNA were observed that were not further elevated upon ARF induction (Figures 4B and 4C). Of note, ARF induction resulted in reduced E2F1 mRNA (Figure S6B) and protein levels (Figure 4B), indicating that E2F1 is prevented from repressing FMN2 by ARF. The ARF-mediated repression of E2F1 has been shown previously to be p53 independent (Mason et al., 2002). This is consistent with our current observations that ARF induction of FMN2 does not depend on p53 expression. We also performed double siRNA knockdown of both E2F1 and NF-κB/RelA and analyzed FMN2 mRNA levels (Figures S6C and S6D). Once again, depletion of E2F1 and NF-κB/RelA resulted in higher levels of FMN2 mRNA, which was not further elevated by ARF induction (Figure S6C).

To further establish the role of NF-κB at the FMN2 promoter, we depleted cells of NF-κB/RelA using siRNA and performed ChIP using both anti-RelA and anti-AcH3 antibodies. Once again, in control cells we could detect binding of NF-κB/RelA to the FMN2 promoter, and this was significantly reduced in cells depleted of NF-κB/RelA (Figure 4D). Importantly, levels of AcH3 present at the FMN2 promoter were increased when NF-κB/RelA was depleted, supporting the previous mRNA and protein expression analysis.

### FMN2 Is Induced by Oncogenic Stress, DNA Damage, and Hypoxia

**Figure 5:**
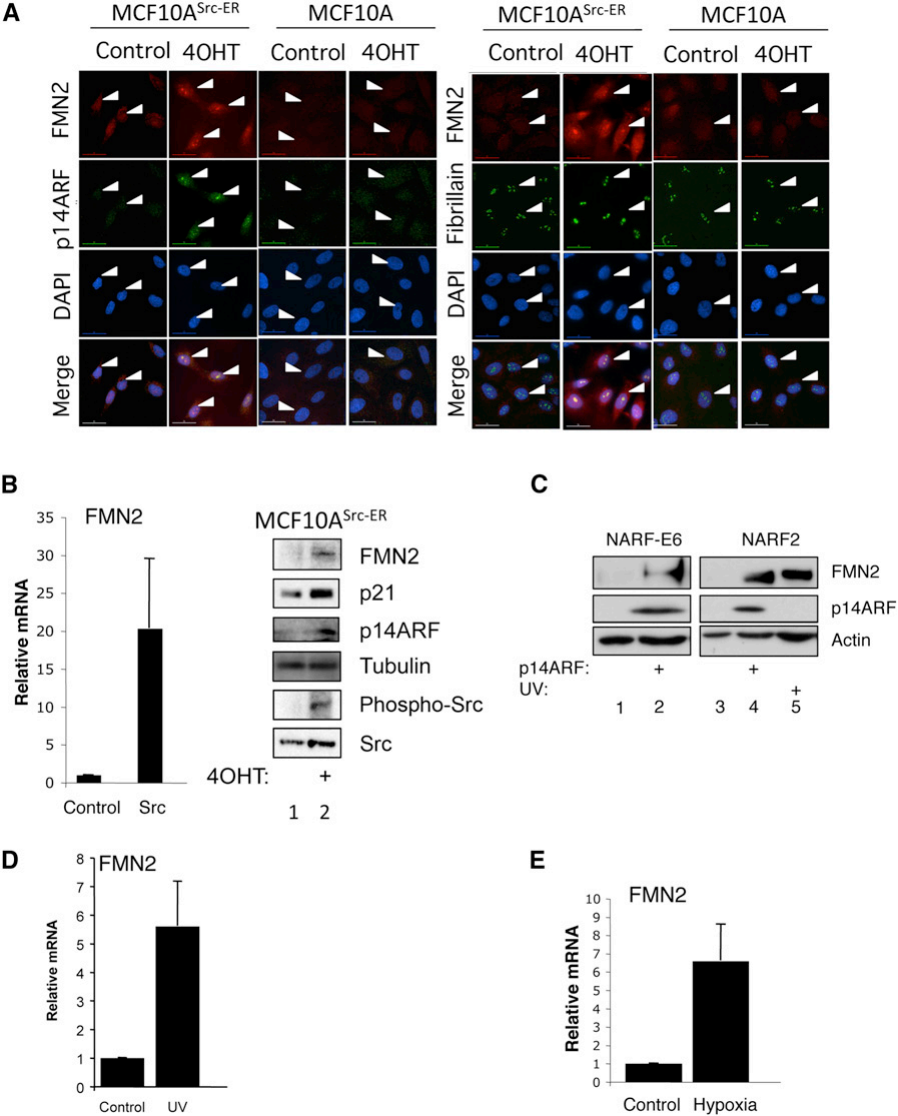
FMN2 Expression Is Induced by Oncogenes, DNA Damage, and Hypoxia (A) Parental MCF10A and Src-inducible MCF10A cells were treated with tamoxifen for 24 hr prior to fixation and immunostaining with the indicated antibodies. (B) Src-inducible MCF10A cells were treated with tamoxifen for 24 hr prior to total RNA (right) or protein (left) extraction. Following cDNA synthesis, qPCR was performed using FMN2-specific primers. Actin was used as a normalizing gene. Graph depicts the mean and standard deviation from a minimum of three independent experiments. Whole-cell lysates were analyzed using the indicated antibodies. (C) NARF-E6 and NARF2 cells were treated with IPTG for 24 hr or UV for 4 hr as indicated prior to lysis. Whole-cell lysates were analyzed by western blot using the indicated antibodies. (D) U2OS cells were treated with UV for 4 hr prior to total RNA extraction. Following cDNA synthesis, qPCR was performed using FMN2-specific primers. Actin was used as a normalizing gene. Graph depicts the mean and standard deviation from a minimum of three independent experiments. (E) U2OS cells were exposed to 1% O_2_ for 24 hr prior to total RNA extraction. Following cDNA synthesis, qPCR was performed and analyzed as in (D). Graph depicts mean and standard deviation from a minimum of three independent experiments.

Given that NF-κB is a transcription factor that responds to many stresses in different cells types (Perkins, 2007), we next determined if the modulation of FMN2 by NF-κB was restricted to ARF induction or was also observed with other stimuli and in different cell types. First, we investigated if activation of the SRC oncogene could lead to changes in FMN2 expression. For this we used a previously described v-SRC-inducible cell system in the breast epithelial MCF10A cells (Iliopoulos et al., 2010). Following induction of v-SRC with tamoxifen, levels of both FMN2 protein and mRNA (Figures 5A and 5B) increased. These results demonstrate that FMN2 levels increase in different cellular backgrounds in response to oncogenic stress, at least under conditions leading to induction of p14ARF.

Next, we investigated if additional stresses would modulate FMN2 expression, independently of ARF. We therefore extended our analysis to examine the effects of DNA damage and hypoxic stress (Figures 5C and 5D), both conditions that have been shown to modulate NF-κB function (Campbell et al., 2004; Culver et al., 2010). In both these situations, FMN2 expression increased. This is illustrated for UV-induced DNA damage by analysis of FMN2 protein (Figure 5C) and mRNA (Figure 5D) levels and for hypoxia by analysis of FMN2 mRNA levels (Figure 5E). These data indicate that FMN2 can respond to several different types of stimuli that all result in the arrest of cell growth.

### FMN2 Controls p21 Protein Levels

**Figure 6:**
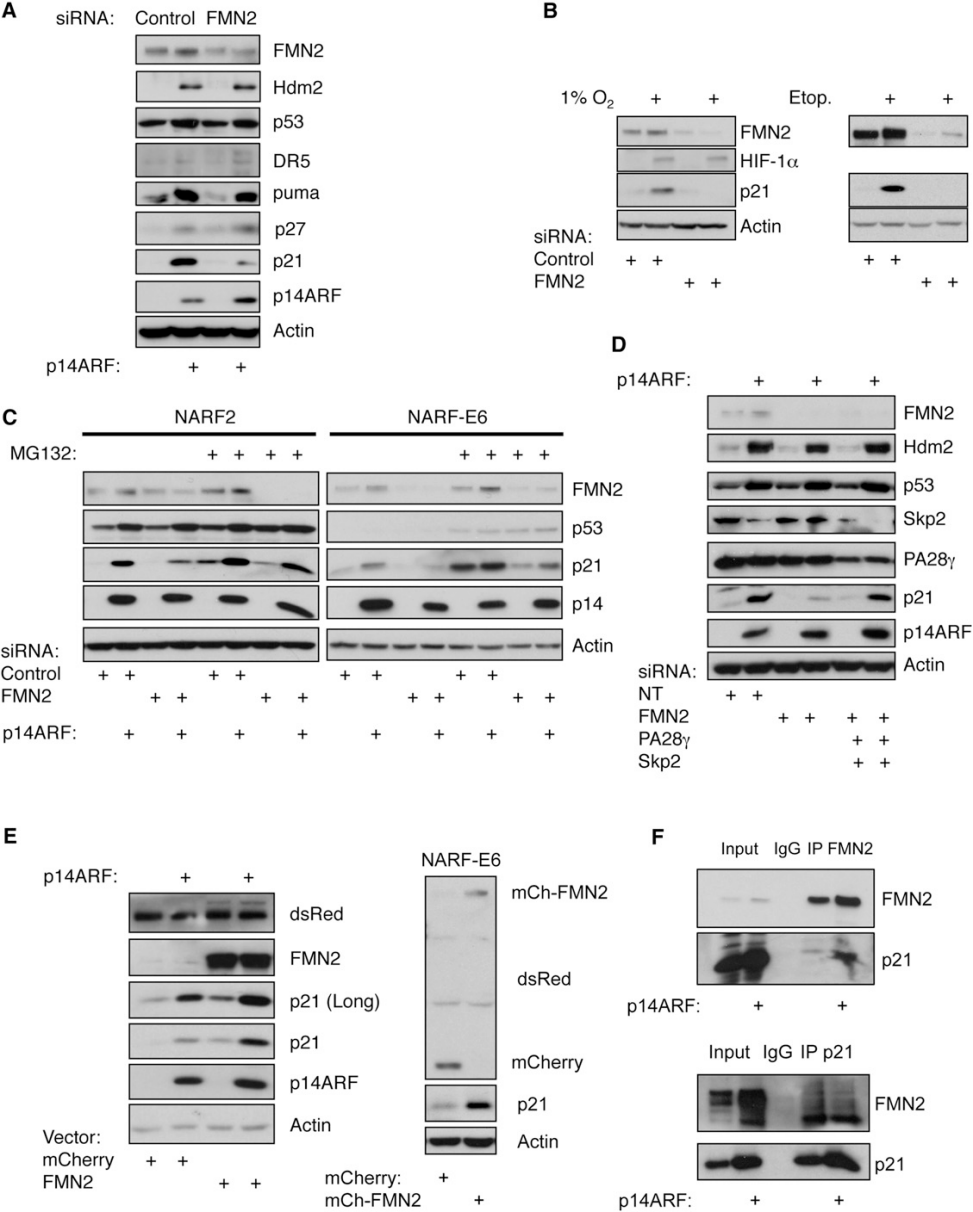
FMN2 Is Necessary and Sufficient for p21 Protein Expression (A) NARF2 cells were transfected with siRNA oligonucleotides for FMN2 prior to IPTG treatment for 24 hr. Whole-cell lysates were analyzed by western blot using the indicated antibodies. (B) U2OS cells were transfected with siRNA oligonucleotides for FMN2 prior to exposure to 1% O_2_ or 10 μM etoposide for 24 hr. Whole-cell lysates were analyzed by western blot using the indicated antibodies. (C) NARF2 and NARF-E6 cells were treated as in (A), but 20 μM of MG132 was added, where indicated, for the last 3 hr of a 24 hr IPTG treatment. Whole-cell lysates were analyzed as in (A). (D) NARF2 cells were transfected with the indicated siRNAs prior to IPTG treatment for 24 hr. Whole-cell lysates were analyzed as in (A). (E) NARF2 and NARF-E6 cells were transfected with 1 μg of empty vector or FMN2 construct. NARF2 cells were also treated or not with IPTG for 24 hr. Whole-cell lysates were analyzed as in (A). (F) NARF2 cells were treated with IPTG for 24 hr prior to lysis. Whole-cell lysates were prepared and immunoprecipitated with normal rabbit IgG or anti-FMN2 (upper panel) or p21 (lower panel) antibodies. Precipitates were resolved by SDS-PAGE and then analyzed by western blotting using indicated antibodies. See also Figure S7.

To investigate the potential functional significance of increased levels of the FMN2 protein in the stress pathways analyzed, we next suppressed FMN2 expression in cells using siRNA (Figure 6). FMN2 protein levels were decreased by the siRNA treatment (Figure 6A). However, we did not observe any changes in the corresponding levels of either p53 or Hdm2 after knockdown of FMN2. Interestingly, however, the levels of the p21 protein were markedly reduced when FMN2 was knocked down (Figure 6A), but not changed when cells were treated with control siRNA (Figure 6A). This effect was prevented by expression of a siRNA-resistant version of FMN2 (Figure S7A), demonstrating that it is the specific change in levels of FMN2 following siRNA treatment that is responsible for altering p21 expression. Levels of p21 protein were also decreased when ARF was knocked down by siRNA (Figure S7B). In contrast, we did not detect any changes in the levels of other known p53 targets, including puma and DR5, after FMN2 knockdown, indicating that FMN2 is not altering general p53 transcriptional activity (Figure 6A).

To examine the mechanism affecting p21 protein levels, we analyzed if p53-mediated induction of p21 mRNA was also altered specifically by FMN2 depletion. We performed qPCR analysis following ARF induction in NARF2 cells, either in the presence or absence of FMN2 knockdown with siRNA (Figure S7C). The results demonstrate that p21 mRNA was induced to similar levels by ARF, regardless of whether FMN2 protein levels were decreased (Figure S7C). As expected, levels of Hdm2, puma, and DR5 mRNA were also unaltered (Figure S7C).

Given that DNA damage and hypoxia also induce p21 protein expression, we next determined if FMN2 was also required for full induction of p21 protein under these types of stress conditions. The results demonstrate that FMN2 is required for p21 protein expression, not only following ARF induction but also following hypoxia and etoposide-induced DNA damage (Figure 6B), indicating a more general role for FMN2 in the control of p21 protein levels. FMN2 depletion by siRNA also resulted in lower p21 protein levels in human foreskin fibroblasts (HFFs) subjected to DNA damage by treatment with etoposide (Figure S7D), demonstrating that this effect is seen in multiple cell types.

The simplest hypothesis explaining these observations is that an increase in FMN2 protein levels is able to stabilize the p21 protein. To test this hypothesis, we depleted FMN2 in NARF2 cells with siRNA, induced ARF expression with IPTG, and added the proteasome inhibitor MG132. The results show that MG132 treatment partially rescues the levels of p21 protein expression after FMN2 depletion (Figure 6C), further suggesting that FMN2 can alter the degree of p21 degradation. Furthermore, this was observed in both NARF2 and NARF-E6 cells, demonstrating the independence from p53 (Figure 6C).

It has been reported that p21 is degraded by both ubiquitin-dependent and -independent pathways (Abbas and Dutta, 2009). To determine which of these pathways are altered by FMN2, we designed siRNAs targeting the proteasome-associated protein PA28γ (Chen et al., 2007; Li et al., 2007) and p21 E3 ligase SKP2 (Frescas and Pagano, 2008), respectively. Knockdown of PA28γ by siRNA partially stabilized p21 protein levels after ARF induction, even when FMN2 was absent (Figure S7E). On the other hand, double depletion of SKP2 and FMN2 also partially stabilized p21 levels after ARF induction (Figure S7E, lanes 11 and 12). Given the partial rescue observed with both PA28γ and SKP2 knockdowns, we next performed a triple siRNA knockdown experiment, where FMN2 was depleted at the same time as both PA28γ and SKP2. Under these conditions, p21 levels were completely rescued following ARF induction. These results strongly suggest that FMN2 functions to stabilize p21 protein levels by preventing its degradation via both ubiquitin-dependent and -independent pathways (Figure 6D).

To further test the model of FMN2 function, we examined the effect of increased levels of FMN2 resulting from transient overexpression of exogenous protein. NARF2 cells were transfected either with an empty vector or with a FMN2-expressing construct, and p14ARF was either induced by the addition of IPTG, or not (Figure 6E). Protein blot analysis demonstrated that even in the absence of p14ARF, exogenous FMN2 protein expression led to increased levels of p21 protein (Figure 6E). This was more pronounced when ARF was induced (Figure 6E). Importantly, increasing FMN2 levels by transient expression did not result in increased levels of p21 mRNA (Figure S7F), further demonstrating the role of FMN2 in posttranscriptional control of p21. Interestingly, an increased basal level of p21 protein is also observed when NF-κB is depleted by siRNA (Figure 4B), a condition that leads to increased FMN2 expression. Stabilization of p21 was also observed in NARF-E6 cells, when exogenous FMN2 was transiently overexpressed (Figure 6E).

To investigate the mechanism by which FMN2 stabilizes p21, we determined if FMN2 was in a complex with p21. To this end, we immunoprecipitated FMN2 and analyzed whether there was coimmunoprecipitation of p21 by western blot. Our analysis revealed that FMN2 and p21 associate in a common complex, both with and without p14ARF induction (Figure 6F, upper panel). Immunoprecipitation of p21 also revealed its association with FMN2 in a common complex (Figure 6F, lower panel). In addition, transient expression analysis of fragments of the full-length FMN2 protein revealed that the N terminus of FMN2 is required for p21 stabilization. Thus, exogenous expression of FMN2 exons 1–5 resulted in higher levels of p21 protein, while exogenous expression of FMN2 exons 6–18 did not (Figure S7G). Taken together, these results indicate that FMN2 may increase p21 protein levels by forming a complex in cells that protects p21 from degradation.

### FMN2 Depletion Induces Apoptosis

**Figure 7:**
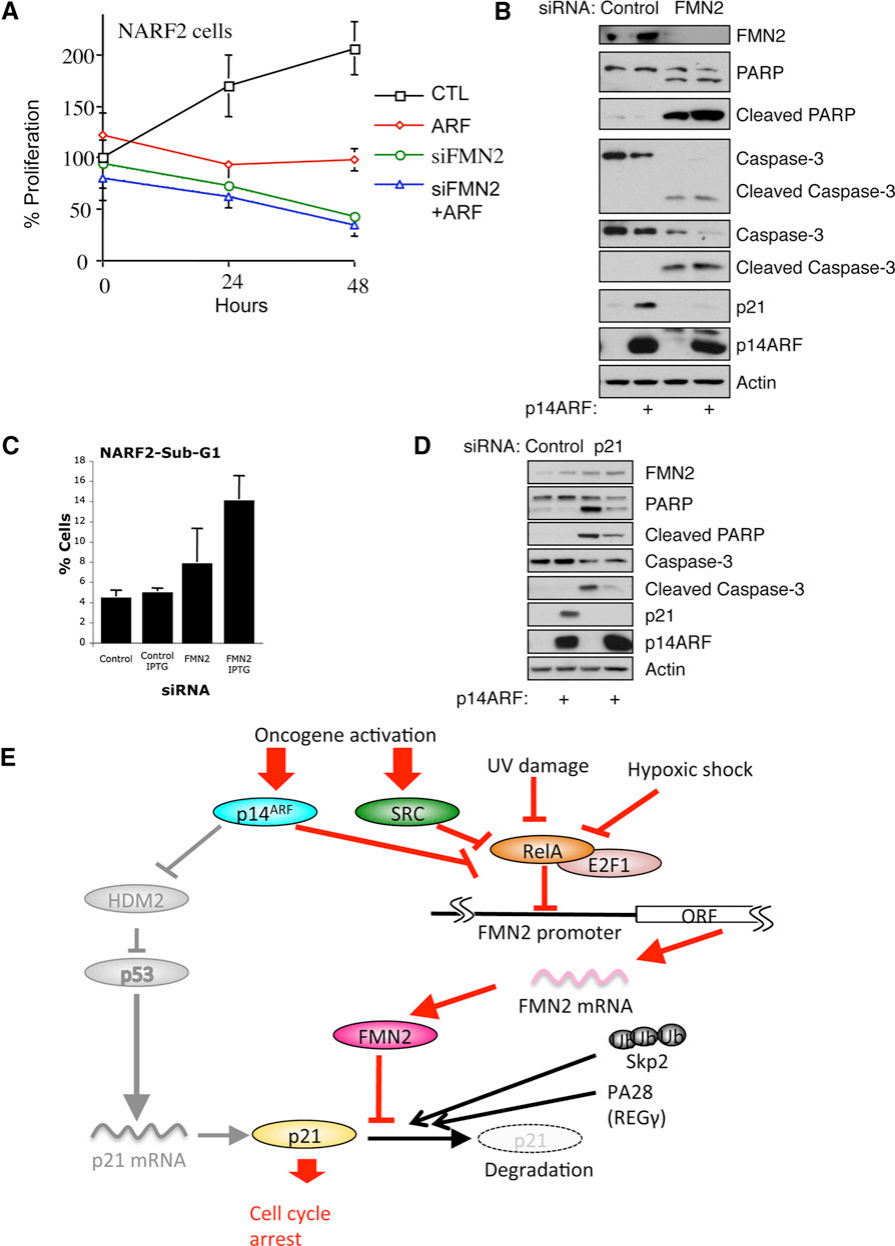
FMN2 Depletion Results in Apoptosis Induction (A) NARF2 cells were transfected with FMN2 siRNAs prior to plating on 96-well plates and IPTG treatment. Proliferation was measured using the Alamar blue assay. Graph depicts the mean and standard deviation of a minimum of three independent experiments performed in triplicate. (B) NARF2 cells were transfected with FMN2 siRNA oligonucleotides prior to IPTG treatment for 24 hr. Whole-cell lysates were analyzed by western blot using the indicated antibodies. (C) Flow cytometry analysis of NARF2 cells transfected with the indicated siRNAs prior to IPTG treatment for 24 hr. Graph depicts percentage of total cells and represents the mean plus standard deviation of a minimum of three independent experiments. (D) NARF2 cells were transfected with p21 siRNA oligonucleotides prior to IPTG treatment for 24 hr. Whole-cell lysates were analyzed by western blot using the indicated antibodies. (E) Schematic diagram depicting our experimental findings. p14ARF, oncogenes, DNA damage, and hypoxia modulate NF-κB and E2F1 to induce FMN2 expression, which is required for p21 protein levels and hence cell-cycle arrest. See also Figure S7.

To determine the functional consequences of the loss of FMN2, and hence reduced p21 levels, in the cellular responses to ARF induction, we analyzed cellular proliferation and viability under conditions in which FMN2 was depleted. ARF induction of p53 can result in either cell-cycle arrest or the induction of apoptosis (Rocha et al., 2005). Most commonly, the initial cellular response is cell-cycle arrest. In NARF2 cells, ARF induction results in stalled proliferation (Figure 7A), with associated cell-cycle arrest (Figure S1A) (Rocha et al., 2005). However, when FMN2 was knocked down by siRNA treatment, cell proliferation was not only stalled, but less viable cells remained compared with the start of the experiment, indicative of cell death (Figure 7A).

Given that ARF induction in the absence of FMN2 still results in the induction of proapoptotic genes, such as puma and DR5 (Figure S7B), we analyzed markers of apoptosis under conditions of FMN2 depletion. The results indicate that after knockdown of FMN2, cells undergo apoptosis, as judged by both caspase-3 activation and PARP cleavage. Interestingly, this response was also observed in the absence of ARF induction, indicating that FMN2 is required for suppression of apoptosis and hence survival of cancer cells (Figure 7B). To examine whether there were defects in cell-cycle progression elicited by FMN2 depletion in combination with p14ARF induction, we performed FACS analysis. These data show that p14ARF induces arrest of cells in both G1 and G2 (Figure S7H), as previously observed (Rocha et al., 2005). FMN2 depletion following p14ARF induction reduces the percentage of cells in G1 but not G2, with a concomitant modest reduction in the number of cells in S phase (Figure S7H). Importantly, depletion of FMN2 with siRNA increases the percentage of cells in sub-G1, indicating an increase in apoptosis (Figure 7C). To determine if this increased apoptosis is due to p21 destabilization, we investigated the effects of p21 depletion following ARF induction. Our analysis revealed that p21 knockdown with siRNA resulted in increased levels of apoptotic markers, including PARP cleavage and caspase activation, consistent with higher numbers of apoptotic cells (Figure 7D). Taken together, these results demonstrate that depletion of FMN2 results in lower p21 protein levels, thereby shifting the cellular response from cell-cycle arrest to apoptosis.

## Discussion

### FMN2 Is Induced Following ARF Induction, Oncogenic Stress, DNA Damage, and Hypoxia

In this study we have identified FMN2 as a key human protein involved in stress-induced cell-cycle arrest. We have shown that FMN2 is critical for p21 protein stabilization but not required for p21 mRNA production. FMN2 levels are upregulated by several different stress stimuli via a common transcriptional mechanism involving NF-κB.

ARF is an important tumor suppressor, acting during oncogene activation (Dominguez-Brauer et al., 2010; Ozenne et al., 2010; Rocha et al., 2005). ARF is a nuclear protein that accumulates in the nucleolus, with a smaller pool also present in the nucleoplasm. This localization is dynamic, and ARF can shuttle between the nucleolus and other nuclear locations. It is best known for its role in binding to and inhibiting Hdm2, the p53 E3 ubiquitin ligase, resulting in stabilization of p53. However, the detailed function of ARF is still not fully characterized. Here we performed a study on nucleolar protein dynamics following a time course of ARF induction in human cells, using quantitative mass spectrometry. We found that ARF induction resulted in the majority of nucleolar proteins decreasing in abundance (Figure 2B). One of the main exceptions was a protein called FMN2, which significantly increased its levels in purified nucleoli following ARF activation.

FMN2 belongs to a family of ubiquitous, conserved multidomain proteins called formins (Faix and Grosse, 2006). Formins are defined by the presence of a formin homology (FH) domain, which confers an actin-nucleating activity to these proteins. FMN2 is expressed in the brain, in the spinal cord, and in oocytes in the mouse. The mouse FMN2 gene has been knocked out and the progeny are viable, but it has been reported that Formin-2-deficient oocytes (*fmn2*^−/−^) do not extrude a first polar body and that they harbor chromosomes that remain most of the time centrally located, suggesting that the first meiotic spindle does not migrate to the cortex in these oocytes (Leader et al., 2002). Interestingly, human FMN2 has relatively low homology with mouse FMN2, with the exception of the FH domain. In particular, the FMN2 N-terminal region has low sequence homology between human and mouse. Of note, despite ARF-mediated stabilization of p53 being conserved in mice, the structure of human p14ARF also differs substantially from the larger mouse ortholog, p19ARF (Ozenne et al., 2010; Wadhwa et al., 2002). Correspondingly, this may suggest that the FMN2 orthologs in human and mouse have evolved with different partner proteins and/or functions in the ARF activation pathway.

Interestingly, our analysis showed that FMN2 is induced not only by ARF, but also by other stresses, including separate forms of DNA damage and hypoxia. The common features in all of these responses appear to be the involvement of NF-κB in the mechanism of transcriptional activation of FMN2 and the downstream effect that they all cause activation of p21 and hence result in an arrest of cell proliferation. The involvement of the FMN2 protein in these responses is an important observation, as is our finding that activation of p21 depends upon actively preventing its rapid degradation and does not result solely from increasing its transcription and translation.

Consistent with our observations in this study, a previous large-scale proteomic analysis of protein targets for phosphorylation by ATR/ATM identified FMN2 as one of multiple targets (Matsuoka et al., 2007). More recently, FMN2 was also reported as a potential oncogene in leukemia (Charfi et al., 2011). Investigation of publicly available data sets in Oncomine (https://www.oncomine.org/resource/login.html) has revealed differential FMN2 RNA expression in human tumors, depending on cancer type. For example, FMN2 RNA is reported to be overexpressed in certain breast cancers and melanomas but underexpressed in certain renal cancers (oncomine). However, the mechanism behind these observations has not been investigated.

Our data demonstrate that under normal growth conditions the transcription factors NF-κB and E2F1 both contribute to repressing FMN2 expression (Figure 4). Specifically, we have demonstrated that NF-κB binds to the FMN2 promoter and is required for transcriptional repression. The NF-κB family of transcription factors, and in particular RelA, has been shown to be activated by stresses such as expression of oncogenes, multiple forms of DNA damage, and hypoxia (Culver et al., 2010; Perkins, 2012). Furthermore, NF-κB, when directly binding to its target promoters, can act as both an activator and a repressor of transcription, depending on posttranslational modifications and association with either coactivators or corepressor proteins (Campbell and Perkins, 2004). Interestingly, NF-κB/RelA and E2F1 have been shown previously to cooperate in the activation of other target promoters, such as EGR1 (Zheng et al., 2009), among others (Lim et al., 2007). Our data now show that NF-κB/RelA and E2F1, which have overlapping binding sites on the FMN2 promoter, can also act to repress transcription, identifying a shared target by these transcription factors. E2F1 and NF-κB proteins are often deregulated in cancer and could account for the lack of FMN2 expression observed in certain cancer types. Further research is necessary to determine if additional control mechanisms are involved in the regulation of FMN2.

### FMN2 Regulates p21 Protein Levels during Oncogene Activation, DNA Damage, and Hypoxia

Our results demonstrate that FMN2 plays an important role in p21 stabilization and reveal that activation of p21 requires a mechanism to actively prevent its rapid degradation. We suggest that this helps to ensure the efficient removal of p21 and prevent its accumulation, except when cells are acutely responding to stress. FMN2 is thus identified as an integral component of the p14ARF-p53 pathway that has a central role in regulating the response to oncogene activation, DNA damage, and hypoxia in human cells. We propose that all stress stimuli that induce cell-cycle arrest via p21 induction may also rely on FMN2 to prevent p21 degradation and hence allow p21 to accumulate to a level where it can promote cell-cycle arrest.

p21 is an important cell-cycle inhibitor, which binds to and prevents the action of cyclin-dependent kinases (Abbas and Dutta, 2009). In addition, it also binds to PCNA and thereby impinges on DNA replication (Li et al., 1994). The p21 protein is a major transcriptional target for the tumor suppressor p53 (Zilfou and Lowe, 2009). Apart from transcriptional control, p21 protein levels are also influenced through both ubiquitin-dependent and -independent degradation pathways (Abbas and Dutta, 2009). Our analysis revealed that FMN2 prevents both degradation pathways from acting on p21 (Figure 6). Indeed, the reduction of p21 levels observed following FMN2 depletion by siRNA could be partially rescued with codepletion of either Skp2 or PA28γ (Figure 6). Importantly, a complete rescue of p21 levels was observed when FMN2 was depleted at the same time as the two different degradation pathways mentioned above. These data indicate that FMN2 is required to protect p21 from the action of pathways that depend on both Skp2 and PA28γ. In addition, we observed that exogenous expression of FMN2 could stabilize the p21 protein, without changing the levels of p21 mRNA. Our analysis revealed that p21 and FMN2 form a complex in cells (Figure 6F). We also found that the N terminus of human FMN2, which is poorly conserved between the human and mouse orthologs, is important for p21 stabilization (Figure S7G). We directly identified peptides from the N terminus of FMN2 in our mass spectrometry analysis. We note that the conserved, actin-binding formin repeats are located in the C terminus of the FMN2 protein. These data indicate an important function for the FMN2 protein that may be independent of the actin binding domain. Indeed, it remains possible that the FMN2 gene could give rise to separate isoforms with distinct functions.

Our data suggest a new extension to the model for ARF tumor suppressor function; thus, while the current model highlights that upon oncogene activation p14ARF-mediated p53 stabilization upregulates p21 mRNA levels, we now show that ARF also transcriptionally upregulates expression of FMN2, independently of p53. In the extended model we present this role of FMN2 as critical to stabilize the p21 protein and allow it to accumulate by preventing its constitutive, rapid degradation (Figure 7E). We propose this revised model involving FMN2 applies generally to the other forms of stress that rely on p21 activation to promote cell-cycle arrest.

Although we focus here on the nucleolar function of ARF, we noticed that FMN2 is also expressed throughout the cell, not only in nucleoli, as observed in a range of different cell types (Figure 5). In this study we have concentrated on characterizing the role of FMN2 in the ARF pathway. In the future, however, it will be interesting to extend this proteomic analysis of ARF to evaluate more broadly downstream effects of ARF induction on protein levels and interactions throughout the cell. A major goal for future studies will now be to investigate in more detail the structure and function of the different domains of FMN2 and how these are regulated in conditions of malignancy.

## Experimental Procedures

### Isolation of Stable Isotope-Labeled Nucleolar Proteins

Cells were grown for at least five cell divisions in either light (Arg0, Lys0), medium (Arg6, Lys4), or heavy (Arg10, Lys8) labeled media before ARF induction (Boisvert et al., 2011). For induction of exogenous p14ARF, IPTG was added at a final concentration of 1 mM to all cells and incubated for 4, 8, 16, and 24 hr, respectively. The experiment was repeated with to give a total of five time points with untreated Arg0, Lys0 cells as a common zero time point. Nucleoli were isolated from NARF2 and NARF2-E6 as previously described (http://www.lamondlab.com/f5nucleolarprotocol.htm). Isolated nucleolar proteins were separated on NuPAGE 4%–12% Bis-Tris gel and excised into 12 slices, and each gel slice was reduced in 10 mM DTT, alkylated in 50 mM iodoacetamide, and subjected to in-gel digestion with trypsin (Andersen et al., 2005). The resulting tryptic pepctides were extracted by 1% formic acid.

### Mass Spectrometry and Data Analysis

Liquid chromatography-tandem mass spectrometry was performed using an Ultimate U3000 nanoflow system (Dionex Corp) and a linear ion trap-orbitrap hybrid mass spectrometer (LTQ-Orbitrap XL, Thermo Fisher Scientific Inc.) via a nanoelectrospray ion source (Proxeon Biosystems) as described previously (Boulon et al., 2010a). Data were acquired usin Xcalibur spftware, and quantification was performed using MS-Quant (http://msquant.sourceforge.net/) and Mascot search engine (Matrix Science) for peptide identification against the International Protein Index (IPI) human protein database. The initial mass tolerance was set to 7 ppm, and MS/MS mass tolerance was 0.5 kDa. Enzyme was set to trypsin/p with three missed cleavages. Carbamidomethylation of cysteine was searched as fixed modification, whereas N-acetyl-protein and oxidation of methionine were searched as variable modification. A minimum of two peptides was quantified for each protein.

### Cells and Transfections

NARF2 and NARF2-E6 cell lines were provided by Dr. Gordon Peters (Cancer Research UK London Research Institute) and have been described previously (Llanos et al., 2001; Rocha et al., 2003; Stott et al., 1998). NARF2 cells, a derivative of the human osteosarcoma U-2OS cells containing an isopropyl β-D-thiogalactopyranoside (IPTG)-inducible p14^ARF^ gene, have been described previously (Stott et al., 1998). The NARF2-E6 cells are a derivative of NARF2 cells but contain, in addition, constitutively expressed human papillomavirus (HPV) E6 protein. MCF-10A and MCF-10A^Src-ER^ were cultured as previously described (Iliopoulos et al., 2010; Schulze et al., 2001). U2OS and HFFs were maintained at 5% CO2 in Dulbecco’s modified Eagle’s medium (Lonza) supplemented with 10% fetal bovine serum (FBS) (Invitrogen), 1% penicillin-streptomycin (Lonza), and 1% L-glutamine (Lonza). All plasmid transfections were performed using effectin (Invitrogen) or GeneJuice (MERCK).

### RNA Isolation and qPCR

Total RNA was extracted using PeqGold total RNA extraction kit (Peqlab) and converted to cDNA using Quantitect Reverse transcription kit (QIAGEN). For quantitative PCR, Brilliant II Sybr green kit (Stratagene/Agilent), including specific MX3005P 96-well semiskirted plates, were used to analyze samples on the Mx3005P QPCR platform (Stratagene/Agilent). Actin was used as a normalizing gene in all experiments. FMN2 expression was analyzed using a one-step Brilliant II Sybr green QRT-PCR mix (Agilent) or using Brilliant II Sybr green kit (Stratagene/Agilent). PCR primers sequences can be found in the Supplemental Information.

### Cell Treatments

Cells were incubated in 1% O_2_ level in an In Vivo 300 hypoxia workstation (Ruskin, UK) for 24 hr. Cells were lysed for protein extracts and RNA extraction in the workstation to avoid reoxygenation. For DNA damage treatments, cells were treated with 10 μM etoposide (Enzo LifeSciences) for 24 hr or exposed to 40 J/m^2^ and harvested 4 hr later. MG132 was purchased from Merck Chemicals and used at the final concentration of 50 μM.

### Microscopy

All cell images were recorded using the DeltaVision Spectris fluorescence microscope (Applied Precision). Cells were imaged using a 60× (NA 1.4) Plan Apochromat objective. Twelve optical sections separated by 0.5 μm were recorded for each field and each exposure (SoftWoRx image processing software, Applied Precision).

### siRNA

siRNA duplex oligonucleotides were synthesized by MWG and transfected using Interferin (Polyplus) as per the manufacturer’s instructions. In brief, cells were plated the day before transfection at the concentration of 2 × 10^5^ cells per well in 6-well plates. The following day, cells were transfected with the final concentration of 5 nM of siRNA oligonucleotides in fresh media, final volume of 2.2 mL. Cells were incubated for additional 48 hr prior to harvesting. IPTG was added for 24 hr unless otherwise stated. siRNA sequences can be found in the Supplemental Information.

### Chromatin Immunoprecipitation

Proteins were crosslinked with formaldehyde for 10 min. Glycine (0.125 M) was added and cells washed with phosphate-buffered saline. Cells were lysed with lysis buffer (1% SDS, 10 mM EDTA, 50 mM Tris-HCL [pH 8.1], 1 mM PMSF, 1 mg/ml leupeptin, 1 mg/ml aprotonin), followed by sonication and centrifugation. The supernatant was precleared with sheared salmon sperm DNA and protein G Sepharose beads (Sigma). The supernatant was incubated with specific antibodies overnight, and then with protein G Sepharose beads for 1 hr. After an extensive wash step, the complexes were eluted with buffer (100 mM NaHCO_3_, 1% SDS) and incubated with Proteinase K. DNA was purified using NBS polymerase chain reaction purification kit (NBS). PCR was performed for the FMN2 promoter.

Antibodies and additional experimental procedures can be found in the Supplemental Information.

## Supplemental Information

Document S1. Figure S1, Figure S2, Figure S3, Figure S4, Figure S5, Figure S6, Figure S7, Supplemental Experimental Procedures, and Supplemental References
