# Supplementary material for: Identification and Functional Characterization of FMN2, a Regulator of the Cyclin-Dependent Kinase Inhibitor p21
Source: Mol Cell. 2013 Mar 7;49(5):922–33. doi: 10.1016/j.molcel.2012.12.023 (PMC3594747; doi:10.1016/j.molcel.2012.12.023)
Supplement: Document S1. Figure S1, Figure S2, Figure S3, Figure S4, Figure S5, Figure S6, Figure S7, Supplemental Experimental Procedures, and Supplemental References [file mmc1.pdf]

Molecular Cell, Volume *49*

Supplemental Information

Identification and Functional Characterization of FMN2, a Regulator of the Cyclin-Dependent Kinase Inhibitor p21

Kayo Yamada, Motoharu Ono, Neil D. Perkins, Sonia Rocha, and Angus I. Lamond

Supplemental Information Inventory

Document containing Supplemental Experimental Procedures, Supplemental References, and Supplemental Figure legends.

Supplemental Figures:

Figure S1 relates to Figure 1

Figure S2 relates to Figure 2

Figure S3 relates to Figure 3

Figure S4 relates to Figure 3

Figure S5 relates to Figure 3

Figure S6 relates to Figure 4

Figure S7 relates to Figure 6 and Figure 7

Fig. S1

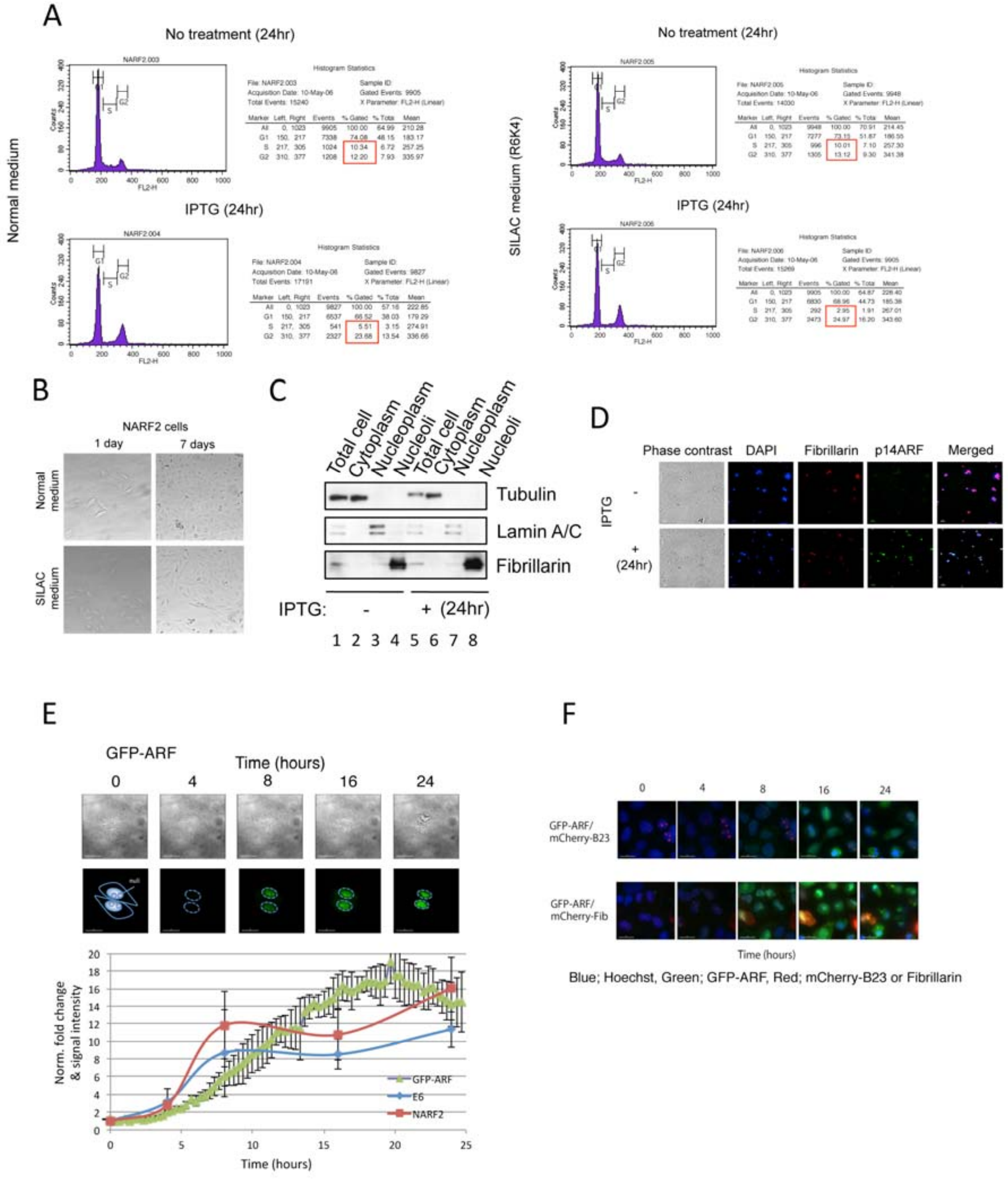

Figure S1, related to Figure 1. (A) NARF2 cells were harvested with or without 24 hours IPTG induction in normal media or SILAC media, and analysed by flow cytometry.

(B) NARF2 cells were cultured 1 to 7 days in normal media or SILAC media, and cell morphology was analysed by microscopy. (C) NARF2 cells were treated with or without IPTG for 24 hours, harvested and fractionated into Nucleoli, Nucleoplasm, and Cytoplasm. Fractions were analysed by western blot using the indicated antibodies. (D) NARF2 were treated and processed as in C. The fractionated Nucleoli from the NARF2 cells were fixed, and immunostained for Fibrillarin (Red), and p14ARF (Green). (E) IPTG inducible GFP-ARF cell line was established, and live cell images were taken for 24 hours following IPTG induction. Green: GFP-ARF, Dot circle: nuclear. Signal intensity has been measured each time point from each cells (n>10) and compared with SILAC ratio obtained from the mass spectrometry analysis. Graph depicts mean and standard deviation from a minimum of 3 independent experiments. (F) mCherry-B23 or mCherry-Fibrillarin plasmid was transfected into GFP-ARF cell line. Live cell images were taken following 24 hours of IPTG induction. Green: GFP-ARF, Red: mCherry-B23 (upper column) or mCherry-fibrillarin (lower column).

Fig.S2

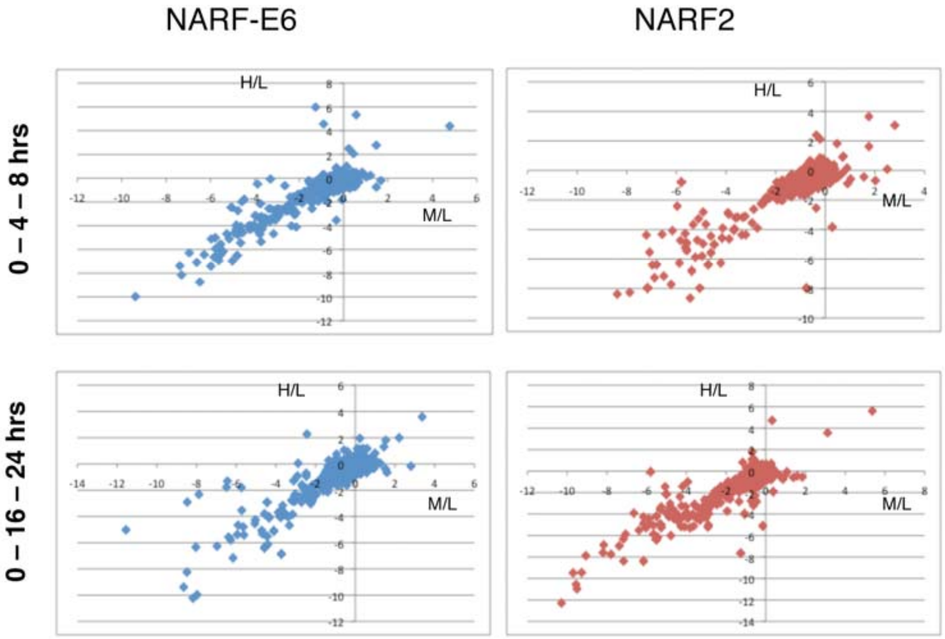

Figure S2, related to Figure 2. Distribution pattern of all the proteins identified during the dynamics of p14ARF activation in NARF2 and E6-NARF2 cell lines. The graph shows Log_2 M/L and H/L ratio in each experiments.

Fig. S3

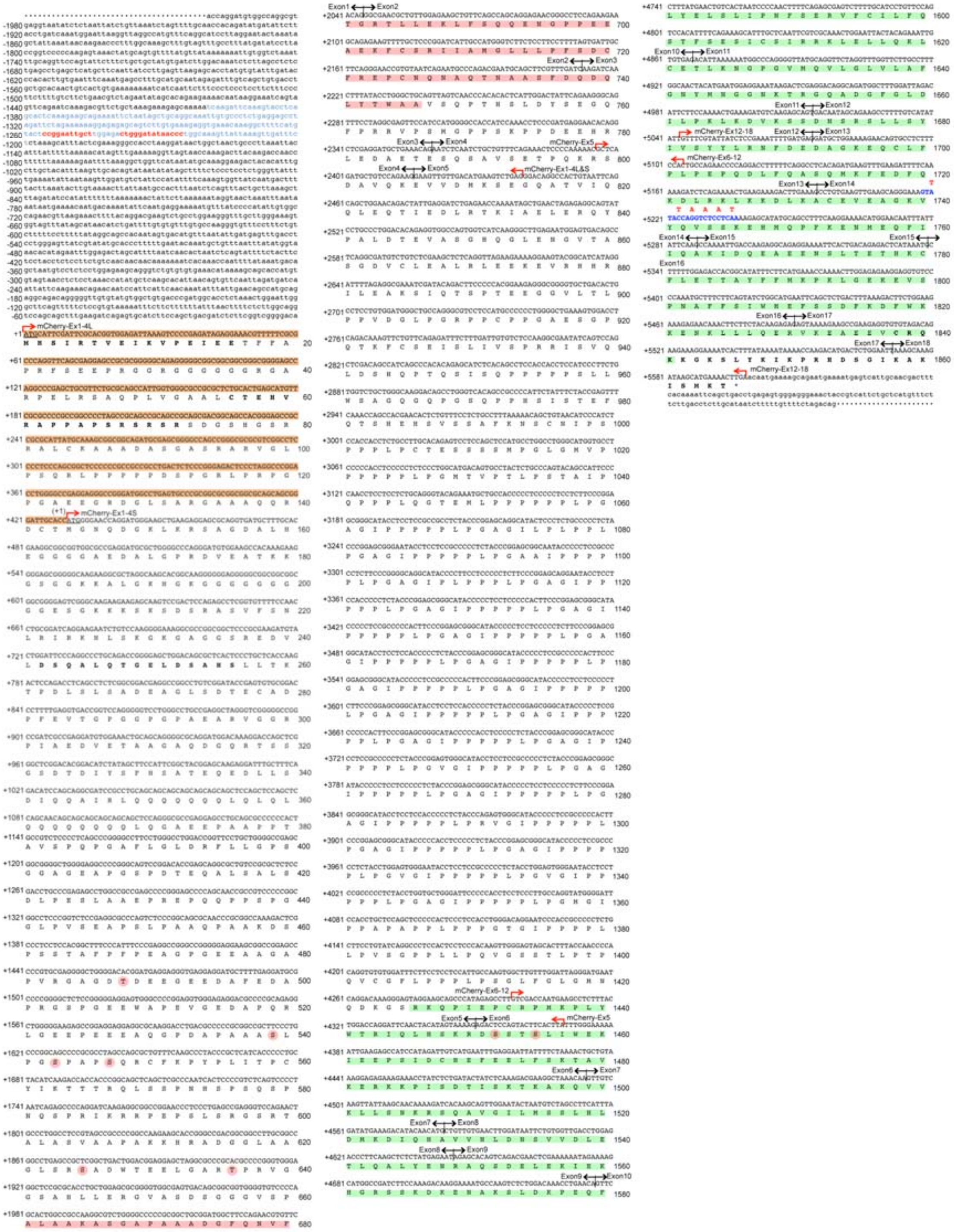

Figure S3, related to Figure 3. FMN2 promoter and coding region sequences. The ARF responsive region, which is located -1400 to -1200 bp is shown in blue. Bold red: putative NF-κB sequences. Orange: Extra N-terminal sequence of FMN2. Bold black: the sequence used for antibody production. Red circle: putative phosphorylation site. Pink: Light green: DEP domain, Bold purple: target sequence for siRNA.

Fig.S4

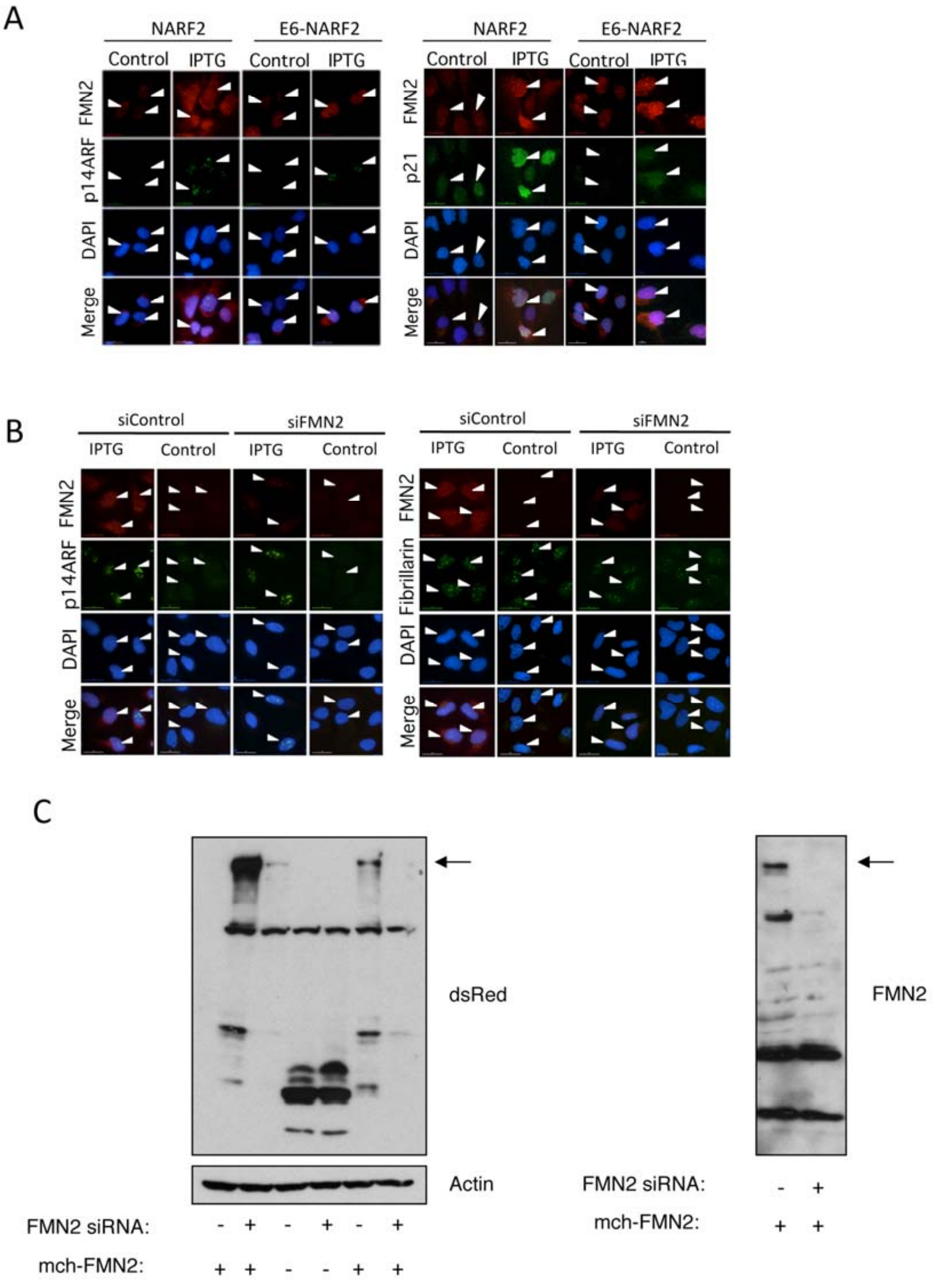
A
NARF2
E6-NARF2
Control
IPTG
Control
IPTG
FMN2
p14ARF
DAPI
Merge
NARF2
E6-NARF2
Control
IPTG
Control
IPTG
FMN2
p21
DAPI
Merge
B
siControl
siFMN2
IPTG
Control
IPTG
Control
FMN2
p14ARF
DAPI
Merge
siControl
siFMN2
IPTG
Control
IPTG
Control
FMN2
Fibrillarin
DAPI
Merge
C
dsRed
Actin
FMN2 siRNA: - + - + - +
mch-FMN2: + + - - + +
FMN2
FMN2 siRNA: - +
mch-FMN2: + +

Figure S4, related to Figure 3. Validation of the FMN2 antibody. (A) NARF2 and E6-NARF2 cells were seeded onto coverslips, treated or not with IPTG for 24 hours prior to fixation. The cells were immunostained for FMN2 and p14ARF. (B) NARF2 cells were seeded as in A, but transfected with FMN2 or control siRNA oligonucleotides, prior to addition or not of IPTG for 24 hours and fixation. Cells were immunostained with the indicated antibodies. Red: FMN2, Green: p14ARF (left column) or Fibrillarin (right column). (C) U2OS cells were transfected with mCherry-FMN2 (full length) expression vector for 24 hours prior to transfection with control or FMN2 siRNA oligonucleotides. Cells were harvested 48 hours later and whole cell lysates were analysed by western blot using the indicated antibodies.

Fig. S5

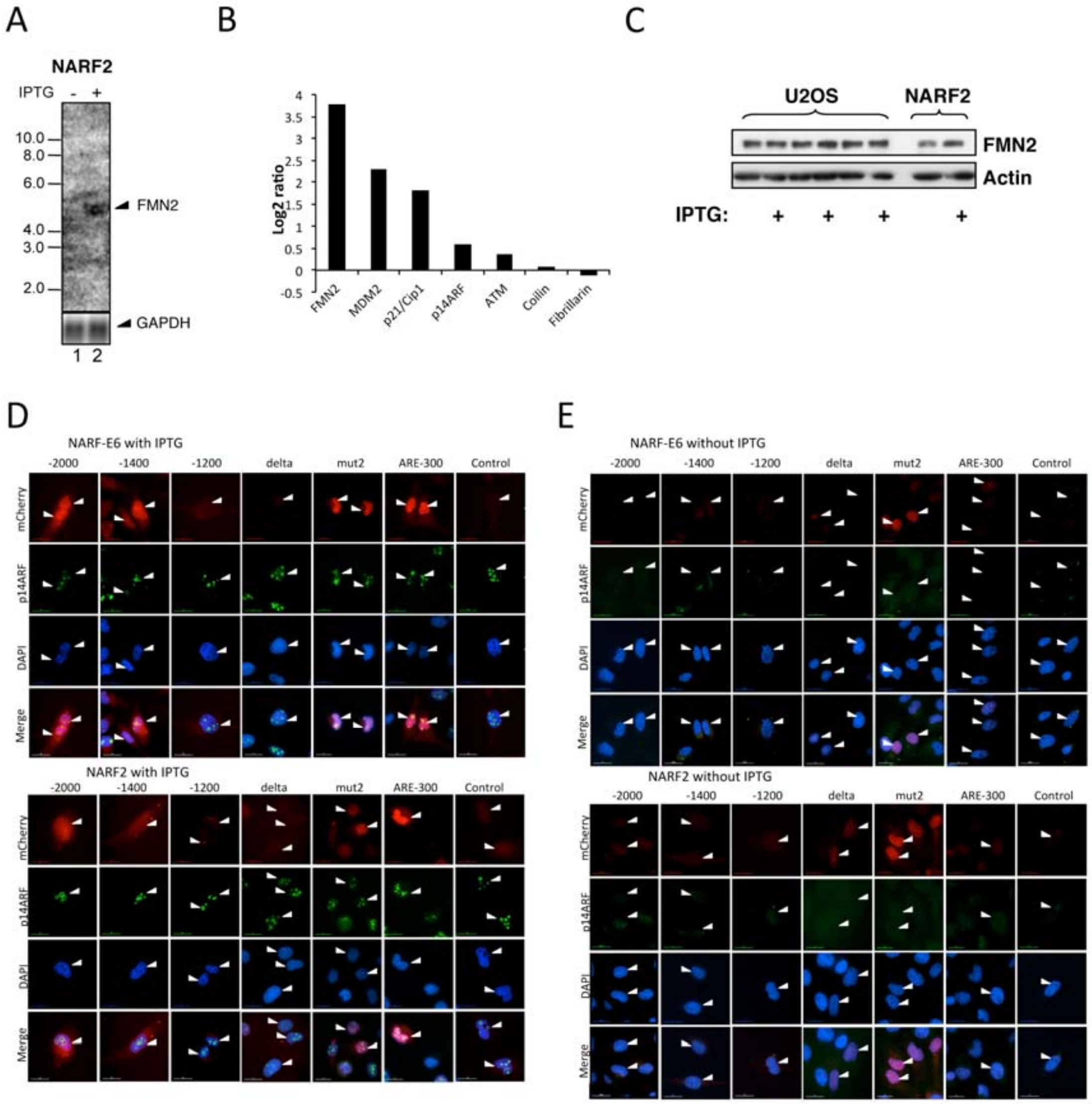

Figure S5, related to Figure 3. FMN2 mRNA is induced by ARF activation. (A) NARF2 cells treated with or without 24 hours IPTG for 24 hours prior to total RNA extraction and northen blot analysis. (B). Cells were treated as in A, but microarray analysis was

performed. Graph depicts results obtained for genes associated to ARF-p53 tumour pathway (MDM2, p21/Cip1, p14ARF, ATM), and also genes not affected by ARF activation inferred from the mass-spectrometry data (Coilin, Fibrillarin). (C) U2OS and NARF2 cells were treated or not with IPTG for 24 hours prior to lysis. Whole cell lysates were analysed by western blot using the indicated antibodies. (D) NARF2-E6 (upper column) and NARF2 cells (lower column) were transfected with pFM-2000, pFM-1400, pFM-1200, pFM_delta, pFM_mut2, pFM_ARE300, or control plasmids, and harvested 48 hours later. Cells were also treated with IPTG for 24 hours. The cells were fixed and immunostained for p14ARF (green), and mCherry fluorescence signals of images were recorded (red). Merged images are combining the p14ARF (green) and mCherry (red) signals. Arrowheads show typical cells. Scale bar 10 μm. (E) NARF2-E6 (upper column) and NARF2 cells (lower column) were transfected with same plasmids described in D and harvested 48 hours but no ARF induction was performed. Arrowheads show typical cells. Scale bar 10 μm.

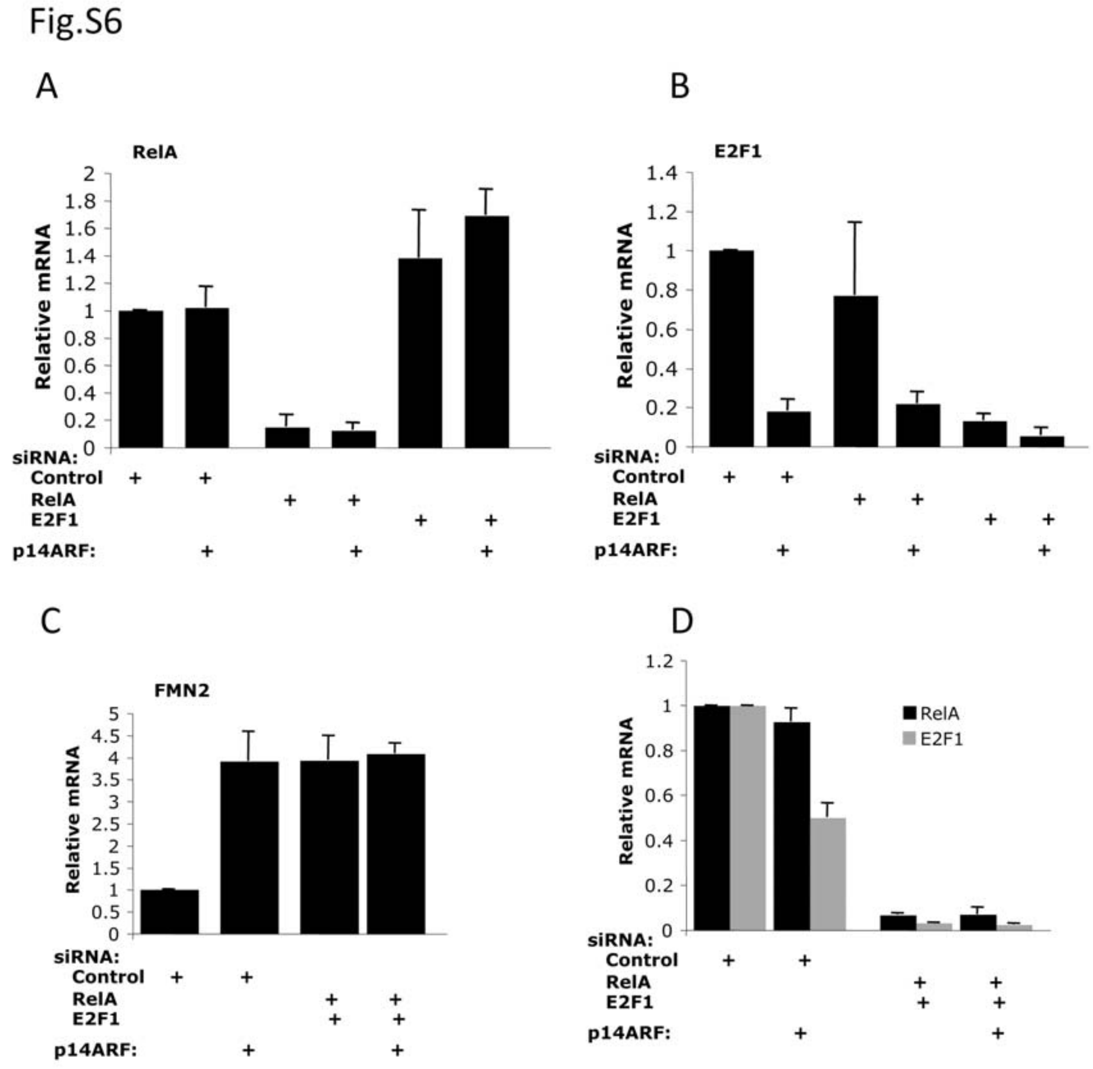

Figure S6, related to Figure 4. FMN2 mRNA is controlled by NF-κB and E2F1. NARF2 cells were transfected with Control, RelA, or E2F siRNA oligonucleotides as indicated and harvested 48 hours with or without IPTG induction for 24 hours. The total

RNA was purified, and analysed by qPCR using RelA (A), or E2F specific primers (B). Graph depicts mean and standard deviation from a minimum of 3 independent experiments. NARF2 cells were transfected with Control, or RelA and E2F siRNA oligonucleotides as indicated, treated or not with IPTG for 24 hours. The total RNA was purified, and analysed by qPCR using FMN2 (C), or RelA or E2F1 specific primers (D). Graph depicts mean and standard deviation from a minimum of 3 independent experiments.

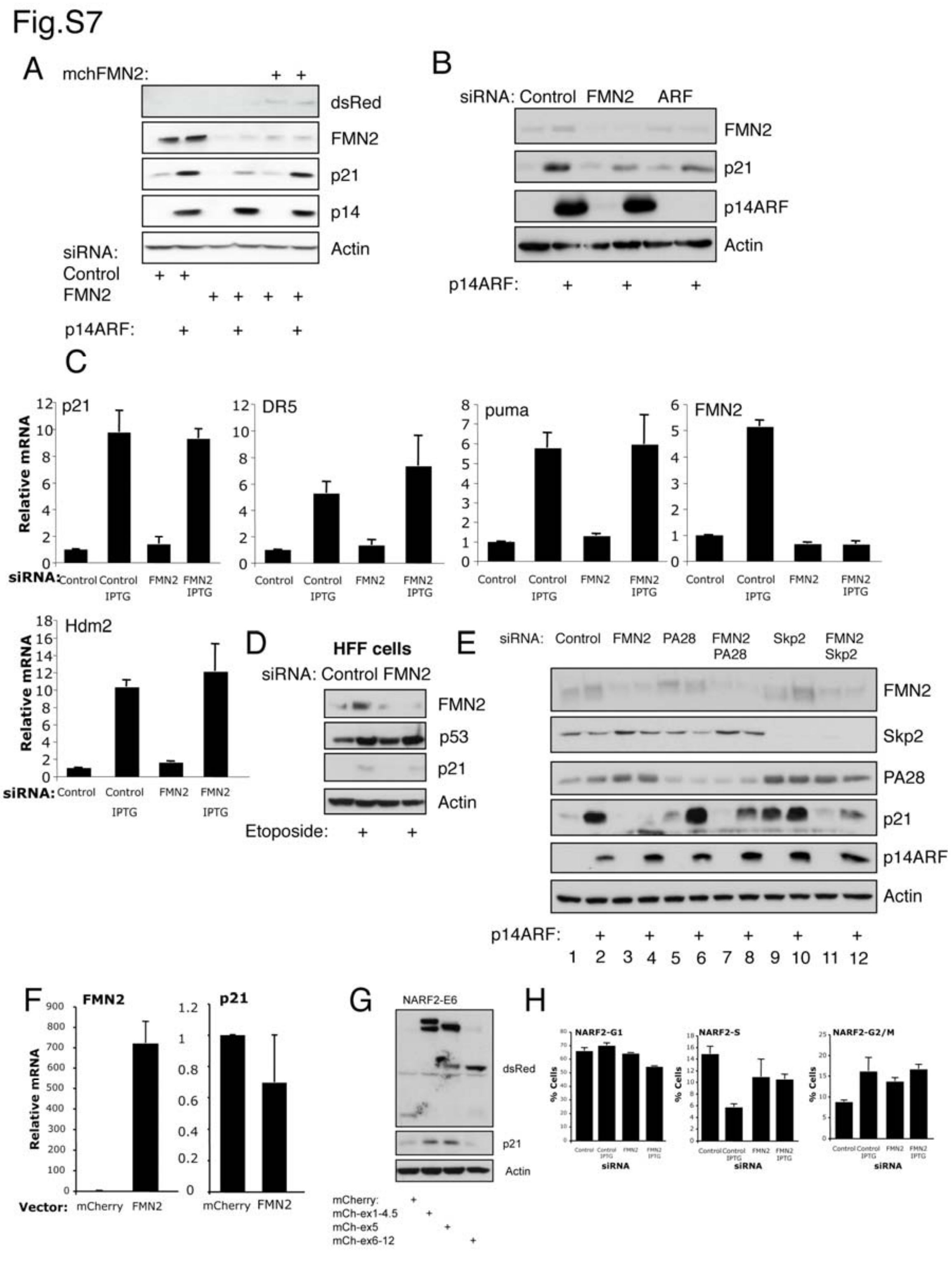

Figure S7, related to Figure 6 and Figure 7. FMN2 is required specifically for p21 protein but not mRNA expression. (A) NARF2 cells were co-transfected with control or

FMN2 oligonucleotides and constructs expressing empty vector or FMN2 siRNA resistant cDNA. Cells were treated or not with IPTG for 24 hours prior to lysis. Whole cell lysates were analysed by western blot using the indicated antibodies. (B) NARF2 cells were transfected with control, FMN2, or p14ARF siRNA oligonucleotides and treated or not with IPTG for 24 hours prior to lysis. Whole cell lysates were analysed by western blot using the indicated antibodies. (C) NARF2 cells were transfected with control or FMN2 oligonucleotides and treated or not with IPTG for 24 hours prior to total RNA extraction. qPCR analysis was performed using DR5, Hdm2, puma or FMN2 specific primers. Graph depicts mean and standard deviation from a minimum of three independent experiments. Actin was used as a normalising gene. (D) HFF cells were transfected with the indicated siRNAs prior to etoposide treatment for 24h. Whole cell lysates were analysed as in A. (E) NARF2 cells were transfected with the indicated siRNAs prior to IPTG treatment for 24h. Whole cell lysates were analysed as in A. (F) U2OS cells were transfected with control or mCherry-FMN2 (full length) expression vector. The total RNA was purified, and analysed by qPCR using FMN2 or p21 specific primers. Graph depicts mean and standard deviation from a minimum of three independent experiments. Actin was used as a normalising gene. (G) NARF-E6 cells were transfected with the indicated expression constructs for 48 hours prior to lysis. Whole cell lysates were analysed as in A. (H) Cell cycle analysis of NARF2 cells transfected with the indicated siRNAs prior to IPTG treatment for 24h. Graph depicts percentage of total cells and represents the mean plus standard deviation of a minimum of three independent experiments.

SUPPLEMENTAL EXPERIMENTAL PROCEDURES

Live cell imaging

GFP-ARF cells were cultured in Willco thin glass-bottomed microwell dishes (Intracel), mounted on a Deltavision Spectris microscope (Applied Precision) fitted in a transparent environmental chamber (Solent Scientific). Cells were imaged 60 x (NA 1.4) Plan Apochromat objective. Twelve optical sections separated by 0.5mm were recorded for each field and each exposure lasted for 0.05 s. After recording the first time points, doxycycline was added at a final concentration of 5ng μg^{-1} and cells were imaged for 2–3 h (SoftWoRx image processing software, Applied Precision). Nucleoli or nuclei (see Fig. 3) were outlined manually and five nucleoli/nuclei were measured from two independent experiments.

Microarray RNA preparation and labelling.

Total NARF2 cell RNA was isolated using the RNeasy mini kit (QUIAGEN), with DNase I treatment, according to manufacturer's instruction. Total RNAs were labelled with One-Color Microarray-Based Gene Expression system (Agilent Technologies).

Microarray experiment and Analysis.

All microarray experiments were performed in EMBL's Genomics Core Facility in Heidelberg, Germany. Labeled cDNA quality were analysed by NanoDrop ND-1000 UV-VIS Spectrophotometer. Labeled cDNA were hybridized on Whole Human Genome Oligo DNA microarray (Human WG 4 X 44k: Agilent Technologies). Scanned data were

analysed by GeneSpring GX software according to manufacture's instruction (Agilent Technology).

Northern blot analysis

Total NARF2 cell RNA was isolated using the TRIzol method, with DNase I treatment, according to the manufacturer's protocol (Invitrogen). Equal amounts of RNA from each sample were separated by 8M Urea polyacrylamide denaturing gel electrophoresis in 1xTBE buffer and the RNA transferred onto nylon membrane (Hybond-N; Amersham) by electro blotting. After UV cross-linking or chemical cross-linking, the membrane was hybridized with ^{32}P 5' labelled partial cDNA probes specific for FMN2 gene.

Plasmids

The FMN2 cDNA was partially amplified from total HeLa RNA. The amplified sequence coding Exon 1 to 4, 6 to 12, and 13 to 18 of FMN2 cDNA were subcloned into mCherry-N1 plasmid and called as pmChe-FMN2Ex1-4, pmChe-FMN2Ex6-12, and pmChe-FMN2Ex13-18, respectively. Because of the amplification inefficiency deriving from the repetitive sequence, the Ex5 sequence was synthesised as oligonucleotides, and subcloned into mCherry-N1 plasmid and called as pmChe-FMN2Ex5. Entire sequence of FMN2 cDNA was subcloned into mCherry-N1 plasmid, and called as pmChe-FMN2full.

For promoter analysis, mCherry-C1 plasmid was removed its CMV promoter region and named as -CMV-mCherry. Predicted FMN2 promoter sequence was generated by PCR from human genome DNA. -1 to -2000 bp upstream from first methionine of

FMN2 gene was cloned into upstream of mCherry sequence in the CMV-mCherry plasmid, and named as pFM-2000. -1 to -1600, -1400, or -1200 bp upstream was cloned into the same region of that plasmid, and named as pFM-1600, pFM-1400, or pFM-1200, respectively. FMN2 cDNA was amplified from HeLa total RNA by RT-PCR, and cloned into mCherry-C1 plasmid. The mutant construct pFM-1600delta was made by deletion of -1400 to -1200 bp FMN2 promoter region from pFM-1600. The pFM-1600mut2 was made by the mutation of two NF-κB binding sites in the pFM-1600 (CCGGAATTGCT to CCAAAAAAACA and CTGGGATATAACCC to CAAAAAAAAAACCC). -1400 to -1200 bp ARF responsive promoter region and -300 to -1 bp basal promoter region was combined and inserted into upstream of mCherry sequence in CMV-mCherry plasmid, and named as pFMARE-300. Detail information about each construct was described in Figure S4.

Primers

Actin

For-CTGGGAGTGGGTGGAGGC

Rev-TCAACTGGTCTCAAGTCAGTG

FMN2

For-ACCGTGTAATCAGAATGCCC

Rev- TCCAAYGAGTGTGTGGGTTG

p21

For- GTCCACTGGGCCGAAGAG

Rev- TGCGTTCACAGGTGTTTCTG

Hdm2

For-CCTGATCCAACCAATCACCT

Rev-TGTTGTGAAAGAAGCAGTAGCA

DR5

For- CTCTCTGCTGGGGAGCTAGG

Rev- AAGACCCTTGTGCTCGTTGT

Puma

For-GTAAGGGCAGGAGTCCCAT

Rev-GACGACTCTAACGCACAGTA

RelA

For-CTGCCGGGATGGCTTCTAT

Rev-CCGCTTCTTCACACACTGGAT

E2F1

For- ATGTTTTCCTGTGCCCTGAG

Rev- ATCTGTGGTGAGGGATGAGG

FMN2-Quantitect Sybr PCR primers (Qiagen)

siRNA sequences

Control-CAGUCGCGUUUGCGACUGG

FMN2-GUAUACCAGGUCUCCUCAA

PA28gamma- GAAUCAAUAUGUCACUCUA

SKP2-ACUCAAGUCCAGCCAUAAG

RelA-GCCCUAUCCCUUUACGUCA

E2F1-CGCUAUGAGACCUCACUGA (Kenneth et al., 2010))

p14ARF-AACAUGGUGCGCAGGUUCUUG (Rocha et al., 2005)

p53; GACUCCAGUGGUAAUCUAC (Schumm et al., 2006).

ChIP primers

FMN2 promoter

FOR-CATTGATGGGAGCCTTAGGA

REV-GAGGACCGTGTGTCATCCTT

Antibodies

Antibodies used were: anti-Actin (Sigma), anti-p21 (Pharmingen and Santa Cruz)), anti-p14ARF (Neomarkers), anti-Hdm2 (Merck Biosciences), Skp2 (Cell Signaling), anti-PA28γ (Pharmingen), anti-PARP (Cell Signaling), anti-cleaved PARP (Cell Signaling), anti-Caspase-3 (Cell Signaling), anti-cleaved Caspase-3 (Cell Signaling), anti-p53 (CR-UK), anti-RelA (Santa Cruz Biotechnology), E2F1 (Cell Signaling), HIF-1α (R&D systems), FMN2 (Abnova), FMN2 antibodies were generated by Dundee Cell Products. Anti-DR5 (Sigma), anti-Tubulin (DM1A, Sigma), anti-Lamin A/C (Santa Cruz), anti-Fibrilarin (72b9, (Reimer et al., 1987)), anti-dsRed (Clonetech), anti-Puma (Cell Signaling).

Antibodies used in ChIPs were:

Acetyl-H3, 1μg (06-599, Millipore), RelA, 2μg (sc-372, Santa Cruz Biotechnology), Rabbit IgG 2μg (Sigma).

Supplemental References

Kenneth, N.S., Mudie, S., and Rocha, S. (2010). IKK and NF-kappaB-mediated regulation of Claspin impacts on ATR checkpoint function. EMBO J *29*, 2966-2978.

Reimer, G., Pollard, K.M., Penning, C.A., Ochs, R.L., Lischwe, M.A., Busch, H., and Tan, E.M. (1987). Monoclonal autoantibody from a (New Zealand black x New Zealand white)F1 mouse and some human scleroderma sera target an Mr 34,000 nucleolar protein of the U3 RNP particle. Arthritis Rheum *30*, 793-800.

Rocha, S., Garrett, M.D., Campbell, K.J., Schumm, K., and Perkins, N.D. (2005). Regulation of NF-kappaB and p53 through activation of ATR and Chk1 by the ARF tumour suppressor. EMBO J *24*, 1157-1169.

Schumm, K., Rocha, S., Caamano, J., and Perkins, N.D. (2006). Regulation of p53 tumour suppressor target gene expression by the p52 NF-kappaB subunit. Embo J *25*, 4820-4832.
